# The human *Cranio Facial Development Protein 1 (Cfdp1*) gene encodes a protein required for the maintenance of higher-order chromatin organization

**DOI:** 10.1038/srep45022

**Published:** 2017-04-03

**Authors:** Giovanni Messina, Maria Teresa Atterrato, Yuri Prozzillo, Lucia Piacentini, Ana Losada, Patrizio Dimitri

**Affiliations:** 1 Istituto Pasteur Italia-Fondazione Cenci Bolognetti and Dipartimento di Biologia e Biotecnologie “Charles Darwin”, Sapienza Università di Roma, Italy; 2 Dipartimento di Biologia e Biotecnologie “Charles Darwin” Sapienza Università di Roma, Roma, Italy; 3 CNIO, Madrid, Spain

## Abstract

The human Cranio Facial Development Protein 1 (*Cfdp1*) gene maps to chromosome 16q22.2-q22.3 and encodes the CFDP1 protein, which belongs to the evolutionarily conserved Bucentaur (BCNT) family. Craniofacial malformations are developmental disorders of particular biomedical and clinical interest, because they represent the main cause of infant mortality and disability in humans, thus it is important to understand the cellular functions and mechanism of action of the CFDP1 protein. We have carried out a multi-disciplinary study, combining cell biology, reverse genetics and biochemistry, to provide the first *in vivo* characterization of CFDP1 protein functions in human cells. We show that CFDP1 binds to chromatin and interacts with subunits of the SRCAP chromatin remodeling complex. An RNAi-mediated depletion of CFDP1 in HeLa cells affects chromosome organization, SMC2 condensin recruitment and cell cycle progression. Our findings provide new insight into the chromatin functions and mechanisms of the CFDP1 protein and contribute to our understanding of the link between epigenetic regulation and the onset of human complex developmental disorders.

Chromatin organization is highly dynamic and subject to many epigenetic changes, mediated by histone modifying enzymes and ATP-dependent chromatin remodeling complexes^1^. These complexes are multi-protein molecular devices able to slide or displace nucleosomes, thus making DNA more accessible to specific binding proteins that control essential cellular processes, such as transcription, replication and DNA repair.

Over the last decade, growing evidence has shown that mutations in genes which encode the epigenetic regulators controlling chromatin configuration can promote cancer and human developmental disorders^2^,^3^,^4^,^5^,^6^,^7^,^8^,^9^. An emblematic case of these “chromatin diseases” is the developmental genetic syndrome called CHARGE^10^, which is caused by mutations in the gene encoding a member of the CHD family of ATP-dependent chromatin remodeling enzymes^4^,^11^. The identification of new candidate genes and proteins will challenge us to expand our understanding of how epigenetic alterations of chromatin structure can perturb development and trigger the onset of human diseases, and will have a significant impact on applied research.

One possible candidate in human developmental diseases is the Cranio Facial Development Protein 1 gene (*Cfdp1*). *Cfdp1* is 139,815 bp long with 7 exons and 6 introns and maps to chromosome 16 in the band 16q22.2-q22.3, where it is proximally and distally flanked by *Bcar1* and *Tmem170A* genes, respectively^12^,^13^,^14^,^15^. *Cfdp1* expression has been detected in a wide range of human tissues, including cancer tissues. It encodes a protein of 299 amino acids, called CFDP1, belonging to the evolutionarily conserved family of Bucentaur (BCNT) proteins^12^,^13^,^14^,^15^. The CFDP1 protein is characterized by an 82-amino acid region located at the C-terminus, called the BCNT-C domain, which is highly conserved among almost all eukaryotes, while the N-terminal region is more divergent^12^,^13^,^14^.

The functions of *Cfdp1* orthologs have been investigated in different species^12^,^13^. In particular, the observation that mouse *Cfdp1* is expressed during tooth development suggested an involvement of this gene in craniofacial development^15^,^16^. Further evidence linked the CFDP1 proteins to craniofacial development and osteogenesis in vertebrates^17^,^18^,^19^,^20^, although specific syndromes caused by mutations of *Cfdp1* have not yet been identified.

An integrative global proteomic study provided evidence suggesting that the CFDP1 protein interacts with members of the SNF2-related CBP activator protein (SRCAP) chromatin remodeling complex^21^ which catalyzes an ATP-dependent exchange of canonical histone H2A for variant H2A.Z in humans^22^. Intriguingly, truncating mutations of the *Srcap* gene cause the rare Floating Harbor syndrome that, among other defects, includes craniofacial abnormalities^9^.

Drosophila YETI and yeast SWC5, two orthologs of CFDP1, were found to be subunits of the d-Tip60 and Swr1 chromatin remodeling complexes, respectively^23^,^24^. Both d-Tip60 and Swr1 complexes share a dozen subunits with the SRCAP complex and are functionally and evolutionarily related to it, in that they also govern variant H2A loading onto chromatin^1^,^23^,^25^,^26^,^27^,^28^.

More recently, it has been shown that the CFDP1 protein expressed in wild-type *Drosophila melanogaster* is able to bind salivary gland polytene chromosomes, strongly affecting chromatin organization and H2Av deposition in a dominant-negative fashion^29^. In addition to its possible role in chromatin remodeling, CFDP1 may also have autonomous functions in transcriptional regulation, as suggested by its interactions with SMAD3 and Ewings Sarcoma (EWS) proteins, which are involved in the modulation of transcription^30^.

Thus far, studies on the *in vivo* function(s) of CFDP1 in human cells are missing.

In the present work, by combining cell biology with reverse genetics and biochemical approaches, we performed a functional analysis of the role played by the CFDP1 protein in human cells. We used Western blotting to detect two endogenous CFDP1 isoforms of 50 and 35 kDa in HeLa, U2OS and MRC5 cell lines. Immunofluorescence microscopy (IFM) and chromatin fractionation assays, together with the expression of intact or truncated Flag-CFDP1 proteins, suggest that the 50 kDa isoform is a chromatin-binding protein that interacts with the SRCAP chromatin remodeling complex. In addition, the depletion of endogenous CFDP1 in HeLa cells drastically affects higher-order chromatin organization and cell cycle progression.

## Results

### Nuclear localization and chromatin association of CFDP1 in human cell lines

**Figure 1:**
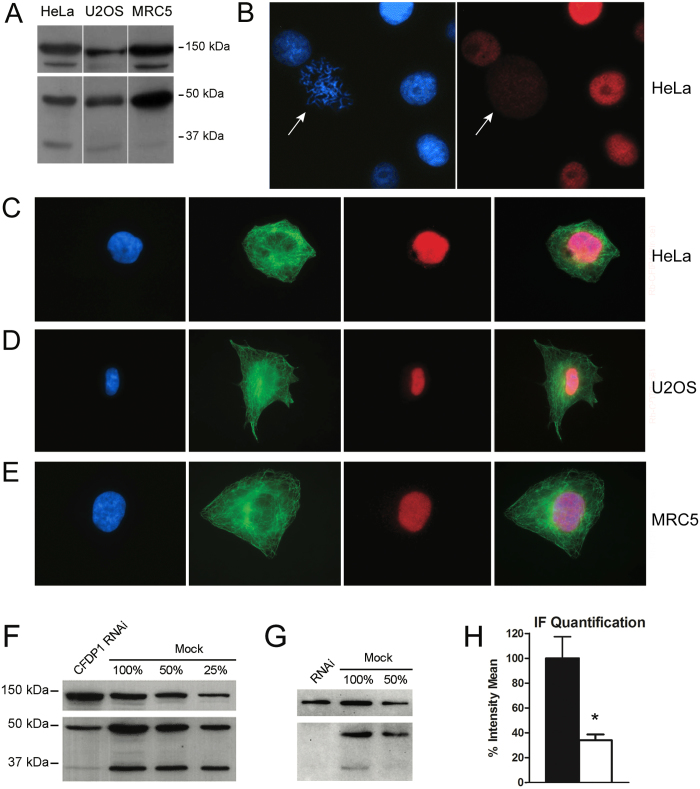
Expression and localization of CFDP1 protein in human cells (**A**) Western blotting of protein extracts from HeLa, U2OS and MRC5 cells with a mouse monoclonal antibody against human CFDP1 (bottom) and ISWI (hSNF2) as loading control (top). The intensity of the 35 kDa band differs between human cells lines tested, being lower in U2OS and MRC5 cells compared to HeLa; this observation suggests that the synthesis of the 35 kDa band (CFDP1 isoform 2) is subjected to regulatory controls. Both 50 kDa and 35 kDa bands are also recognized by a rabbit polyclonal antibody to CFDP1 (see G). (**B**) Fixed HeLa cells were stained with the mouse monoclonal antibody to CFDP1 (red) and counterstained with DAPI (blue). The CFDP1 staining is clearly present in the interphase nuclei, while no significant staining was detected on mitotic chromosomes (pointed by arrows). (**C**,**D**,**E**) Human cell lines were also incubated with a rabbit polyclonal antibodies to human CFDP1 and with commercial antibodies to α-tubulin. From left to right panels: DAPI (blue), α-tubulin (green), CFDP1 (red) and merge in: (**C**) HeLa, (**D**) U2OS and (**E**) MRC5 cells. A significant fluorescent staining of interphase nuclei was observed in all the three lines. (**F**) and (**G**) Western blotting with mouse monoclonal and rabbit polyclonal antibodies to CFDP1, respectively; it appears that in RNAi-treated cells (RNAi lane) the intensity of both CFDP1 bands is strongly reduced compared to the mock-treated control cells (mock 100% lane); ISWI (hSNF2) was used as control (top). (**H**) The CFDP1 nuclear staining with monoclonal antibodies shows about 70% decreased in RNAi-treated HeLa cells compared to the mock.

We initially performed Western blotting assays on total protein extracts from HeLa, U2OS and MRC5 cell lines, using a mouse monoclonal antibody to CFDP1 (see Materials and Methods). The results showed the presence of two sharp bands of about 50 kDa and 35 kDa in all three cell lines (Fig. 1A). The *Cfdp1* gene is predicted to undergo alternative splicing, giving rise to two mRNAs which differ for the presence or absence of the last exon (http://www.uniprot.org/blast/?about=Q9UEE9-2). It is conceivable that the 50 kDa and 35 kDa bands represent the CFDP1 isoforms 1 and 2, respectively, the shorter isoform lacking the last 82 amino acids which correspond to the evolutionary conserved BCNT domain.

Next, we used immunofluorescence microscopy (IFM) with the same monoclonal antibody to visualize the cellular localization of the endogenous CFDP1. As shown in Fig. 1B, CFDP1 is present in the nucleus of HeLa cells in interphase but is not seen on mitotic chromosomes. The staining of interphase nuclei was confirmed by additional IFM experiments performed in HeLa, U2OS and MRC5 cell lines using a rabbit polyclonal antibody to CFDP1 (Fig. 1C,D,E; see Materials and Methods). To validate the specificity of the antibodies to CFDP1, Western blotting and IFM experiments were performed after RNAi-mediated depletion of CFDP1 in HeLa cells. As shown in Fig. 1F,G,H, the amount of CFDP1 was lower in RNAi-treated cells compared to mock-treated cells.

**Figure 2:**
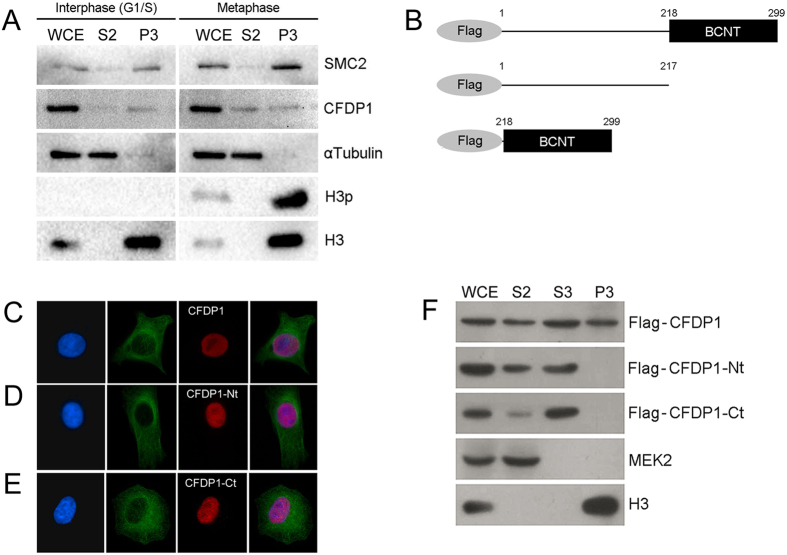
Chromatin association of CFDP1 in HeLa cells (**A**) Western blot analysis of fractionated HeLa cells after synchronization in interphase (G1/S) or metaphase (see material and methods). As metaphase synchronization controls, the histone 3 phosphorylated in serine 10 (H3p) and SMC2 condensin have been used. H3p is a DNA condensation marker which is found only in mitotic chromatin, while SMC2 increases in condensed chromatin. As expected, H3p is specifically present in the chromatin-bound fraction (P3) of HeLa cells synchronized in metaphase. In the same fraction, the amount of SMC2 is higher compared to that of cells synchronized in G1/S. CFDP1 is detected at comparable amount in the chromatin-bound fractions (P3) of both interphase and metaphase, similar to histone H3. (**B**) Schematic representation of Flag-CFDP1 tagged proteins; Flag-CFDP1 full-length, isoform 1(aa 1-299); Flag-CFDP1-Nt, isoform 2 (1–217) and Flag-CFDP1-Ct, BCNT domain (218–299). (**C**) From left to right panels: DAPI (blue), α-tubulin (green), Flag-CFDP1 (red) and merge. (**D**) From left to right panels: DAPI (blue), α-tubulin (green), Flag-CFDP1-Nt (red) and merge. (**E**) From left to right panels: DAPI (blue), α-tubulin (green), Flag-CFDP1-Ct (red) and merge. (**F**) Western blot analysis of fractionated HeLa cells after transfection with Flag-CFDP1 or truncated variants. Flag-CFDP1 is detected in soluble (S2 and S3) and chromatin-bound (P3) fractions, while Flag-CFDP1-Nt and Flag-CFDP1-Ct are found only in the soluble fractions (S2 and S3). MEK2 and H3 are specific controls for soluble and chromatin fractions, respectively. Anti-MEK2, mitogen-activated protein (MAP)/ERK *kinase* 2, was used as cytoplasmic contamination control in P3 fraction; anti-histone H3 was used to monitor chromatin contamination in soluble fraction. WCE = whole cell extract; S2 = cytoplasmic soluble fraction; S3 = nuclear soluble fraction, P3 = chromatin-bound fraction.

The absence of anti-CFDP1 staining on chromosomes (Fig. 1B) may suggests that CFDP1 is not bound to chromatin in mitosis, similarly to what has been found for HMG-14/-17 proteins^31^. Alternatively, CFDP1 could also be present in mitotic chromatin, but less accessible to the antibody. To discriminate between these two hypotheses, we performed a fractionation assay on HeLa cells synchronized in interphase (G1/S) or metaphase. As shown in Fig. 2A, the 50kDa band of CFDP1 is found at comparable amounts in the chromatin-bound fraction (P3) of both interphase and metaphase cell extracts. In summary, CFDP1 is expressed in human cells of different origins, probably as two differently spliced variants and is bound to chromatin in both interphase and metaphase, although is not visualized on chromosomes by immunostaining.

### Chromatin association of CFDP1 requires both N- and C-terminal regions

To further validate the nuclear localization and chromatin binding ability of CFDP1, as is the case for the *Drosophila melanogaster* ortholog YETI^24^, we expressed flag-tagged CFDP1 from a full length cDNA and two truncated cDNAs that carried either the N-terminal or the C-terminal portion of the gene, in HeLa cells (see Materials and Methods). Worth noting, the Flag-CFDP1-Nt variant exactly matches the putative isoform 2 of CFDP1, in that it carries the first 217 amino acids and lacks the C-terminal BCNT domain (Fig. 2B).

**Figure 3:**
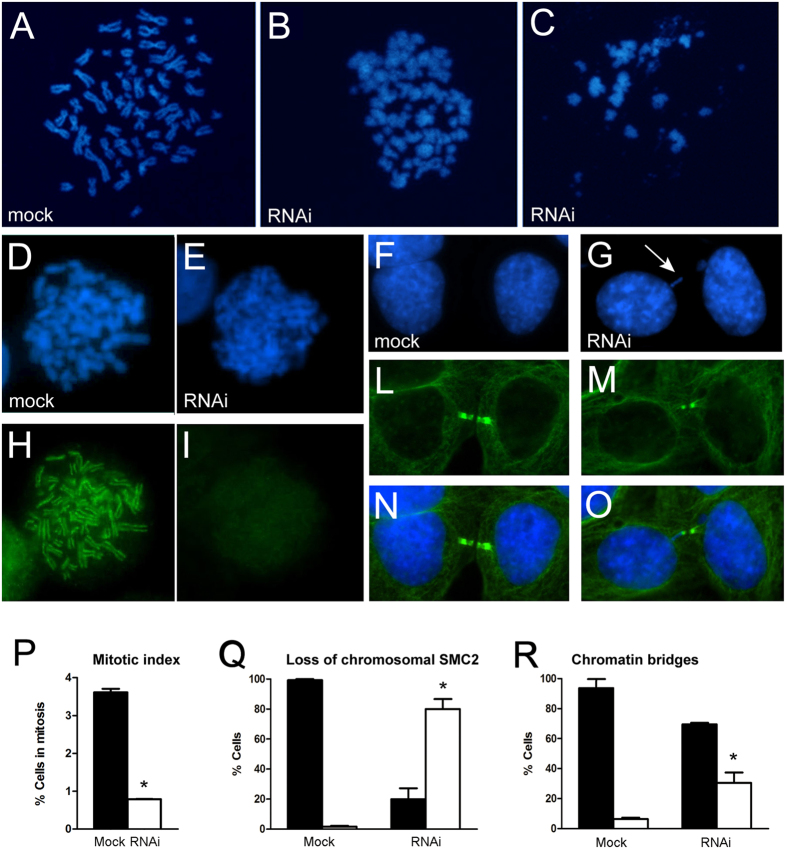
Mitotic defects after RNAi depletion of CFDP1 in HeLa cells (**A**) Metaphase chromosomes from mock-treated HeLa cells; (**B**) and (**C**) Clearly aberrant metaphase chromosomes that fail to undergo proper condensation found in RNAi treated HeLa cells; condensing chromatin appeared fuzzy, loose and largely disorganized. Hela cells were treated with Colcemid to enrich for metaphase chromosomes. (**D**) and (**E**) DAPI and SMC2 condensin sequential staining of mock treated cells; the SMC2 staining decorates the chromatid axes (**H**) and (**I**) DAPI and SMC2 condensin sequential staining of CFDP1 depleted cells; the SMC2 staining is very faint. (**F**) and (**L**) DAPI and α-tubulin staining of telophases in mock-treated cells; (**N**) Merge; (**G**) and (**M**) DAPI and α-tubulin staining of telophases in CFDP1 depleted cells; (**O**) Merges. The arrow in (**G**) points an example of chromatin bridge. (**P**) Quantification of the mitotic index of CFDP1 depleted cells compared to the mock. The mitotic index is expressed as the percentage of the total population of cells in mitosis (*n* = 1000). Cell cycle stages were determined by scoring all mitotic cells by IF for α-tubulin and chromosome staining; black and white histograms represent % of cells in mitosis in mock and CFDP1-depleted cells, respectively. (**Q**) Quantification of loss of chromosomal SMC2 in CFDP1-depleted cells compared to the mock; black and white histograms represent % of cells with normal or aberrant distribution of SMC2 respectively. (**R**) Quantification of telophases showing chromatin bridges in CFDP1 depleted cells compared to the mock. About 30% of telophases show the occurrence of chromatin bridges. Black and white histograms represent % of normal and aberrant telophases in mock and CFDP1-depleted cells, respectively.

IFM showed that Flag-CFDP1, Flag-CFDP1-Nt and Flag-CFDP1-Ct are all able to enter the cell nuclei (Fig. 3C,D,E). A fractionation assay, however, showed all the three forms present in the soluble fractions, while only Flag-CFDP1 is detectable in the chromatin-bound fractions (Fig. 2F). It appears that both Flag-CFDP1-Nt and Flag-CFDP1-Ct truncated forms are defective for the chromatin binding activity. We must conclude that two different regions of CFDP1, one included in Flag-CFDP1-Nt and the other in Flag-CFDP1-Ct, are present simultaneously for chromatin binding. In addition, these results, together with the results obtained in synchronized HeLa cells (Fig. 2A), strongly suggest that the chromatin-binding activity is specific to the 50 kDa isoform of CFDP1, while the 35 kDa isoform may have different functions.

### Depletion of CFDP1 in HeLa cells affects chromosome organization and condensin recruitment in mitosis

To investigate the function of CFDP1 protein in human cells, we performed a cytological analysis of fixed HeLa cell preparations after RNAi-mediated knock-down of *Cfdp1* (see Materials and Methods). The efficiency of CFDP1 protein depletion was monitored by Western blotting, as previously shown in Fig. 1F,G. Metaphase chromosomes from CFDP1-depleted cells displayed aberrant morphology and condensation defects when compared to mock-treated cells (Fig. 3A–C). In a total of 115 scored metaphase chromosome spreads, 45% of abnormally condensed figures were seen in *Cfdp1*-siRNA treated cells, while only 3% of such metaphases were found in the mock-treated controls. Thus, it appears that CFDP1 activity is required for the maintenance of proper higher-order chromatin organization in human cells. This behavior strongly resembles that of the *Drosophila* YETI protein^24^. In addition, the count of mitotic figures showed that the mitotic index in CFDP1 depleted cells is decreased about 75% compared to controls (Fig. 3P), indicating that the loss of CFDP1 can also affect cell cycle progression.

Given that the condensin complexes play a key role in chromosome condensation, we also studied the localization of SMC2, a subunit of the condensin I and II complexes^32^ in CFDP1-depleted HeLa cells. The results of this analysis are shown in Fig. 3D,E,H,I. In about 80% of metaphase cells (634 in total) a significant loss of chromosomal SMC2 was detected (Fig. 3Q). We also observed a high proportion (30%) of telophases with chromatin bridges (Fig. 3F,G,L,M,N,O,R). This abnormality may be the consequence of chromosome stickiness due to condensation defects. Together, these results strongly support a role for CFDP1 in chromatin organization.

### Expression of Flag-YETI in HeLa cells mimics CFDP1 depletion

**Figure 4:**
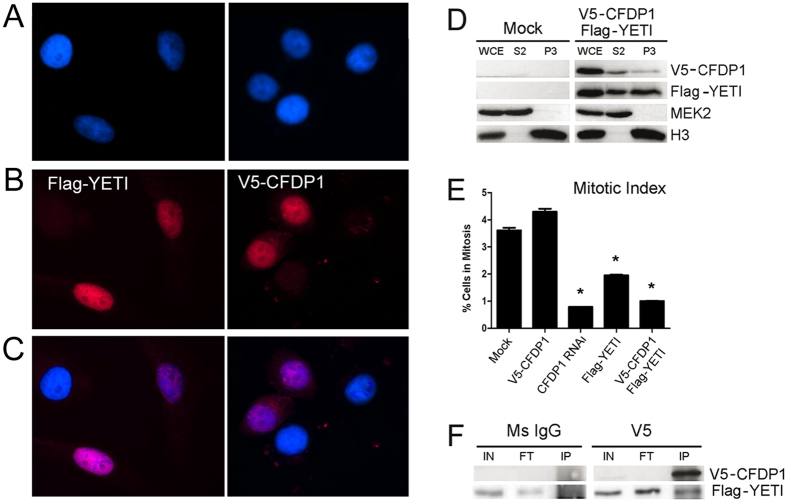
Drosophila YETI impairs CFPD1 function in HeLa cells (**A**) DAPI staining of HeLa cells expressing Flag-YETI (left panel) or V5-CFDP1 (right panel); (**B**) Nuclear localization of V5-CFDP1 (left panel) and Flag-YETI (right panel) proteins. (**C**) Merges; (**D**) Western blot analysis of fractionated HeLa cells after V5-Cfdp1/Flag-Yeti transfection. WCE, whole cell extract; S2, cytoplasmic soluble fraction; P3, chromatin-bound fraction. (**E**) Analysis of mitotic index of HeLa cells. HeLa cells overexpressing V5-CFDP1 fusion protein slightly increases mitotic index, compared to the mock-transfected control. RNAi-mediated depletion of CFDP1 produces about 75% reduction of mitotic index; FLAG-YETI overexpression causes a 60% reduction of mitotic index; (**F**) For IP assays, protein lysate was prepared from HeLa expressing V5-CFDP1 and FLAG-YETI fusion proteins and immunoprecipitated using a mouse anti-V5 antibody. A FLAG-YETI band is detected in the V5-immunoprecipitate, but not in Ms IgG negative control.

Our previous results suggest that when CFDP1 is expressed in wild-type fruit flies, it can physically interact with YETI to form inactive heterodimers; this would result in an overall depletion of functional YETI with a consequent disruption of chromatin organization and individual viability^29^. To test whether YETI overexpression may affect cell behavior in human cells, we transiently transfected HeLa cells with Flag-*Yeti* and V5-*Cfdp1* cDNA constructs. The IF staining showed that V5-CFDP1 and Flag-YETI are able to enter the nuclei (Fig. 4A–C). Moreover, in a fractionation assay both Flag-YETI and V5-CFDP1 were found in the chromatin-bound fraction, as well as in the soluble fraction (Fig. 4D). As shown in Fig. 4E, HeLa cells overexpressing Flag-YETI exhibit a significant decrease in mitotic index (about 50%), although the effect is not as strong as in CFDP1 depleted cells. By contrast, the overexpression of the V5-CFDP1 fusion protein did not affect mitotic index. In addition, in HeLa cells overexpressing both Flag-Yeti and V5-*Cfdp1* constructs, the V5-CFDP1 fusion protein is not able to rescue the mitotic index decrease (Fig. 4E). To test whether CFDP1 and YETI interact *in vivo*, immunoprecipitation assays were performed in HeLa cells transfected with both V5-*Cfdp1* and Flag-*Yeti* constructs. As shown in Fig. 4F, FLAG-YETI fusion protein was detected in the IP sample immunoprecipitated with V5 antibody, but not in IgG negative control. Together, these findings suggest that CFDP1 and YETI can physically interact *in vivo*, giving rise to inactive heterodimers that can affect cell viability.

### Co-IP assays in HeLa cells

As discussed in the introduction, a global proteomic study provided evidence that the CFDP1 protein interacts with members of the SNF2-related CBP activator protein (SRCAP) chromatin remodeling complex^21^. However, thus far these data have been not validated *in vivo*. To test whether CFDP1 interacts *in vivo* with members of the SRCAP complex, we performed a series of co-IP assays. HeLa cells were transfected with 3 different expression vectors containing different tagged proteins: CFDP1 tagged with V5 (V5-CFDP1), H2A.Z tagged with HA (HA-H2A.Z) and Arp6 tagged with Myc (Myc-Arp6). In parallel, HeLa cells were also transfected with two expression vectors containing CFDP1 tagged with V5 (V5-CFDP1) and SRCAP tagged with HA (HA-SRCAP). Cell extracts were immunoprecipitated with anti-V5 antibodies and then analyzed by Western blotting using antibodies against anti-Myc, HA and other known components of the SRCAP complex. As a negative control, we used HeLa cell extracts immunoprecipitated with Ms IgG.

**Figure 5:**
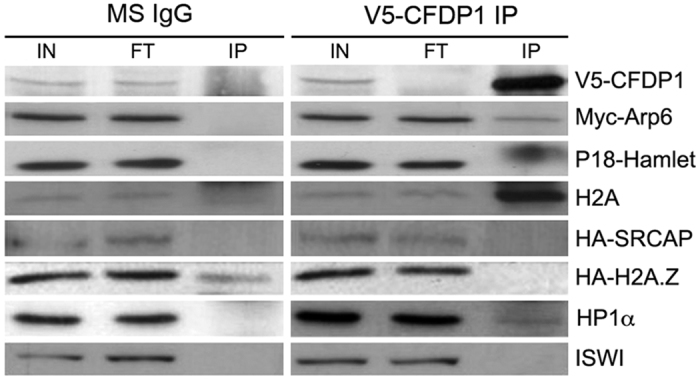
CFDP1 interacts with subunits of the SRCAP complex Total protein extracts from HeLa cells expressing V5-CFDP1, Myc-Arp6 and HA-H2A.Z or V5-CFDP1 and HA-SRCAP vectors were subjected to immunoprecipitation with the anti-V5 antibody. Input protein extracts (IN), flow-through (FT) and V5 immunoprecipitates (IP) were separated by SDS–PAGE and transferred onto a nitrocellulose membrane. Western blots were probed with α-V5, α-Myc, α-P18^Hamlet^, α-H2A, α-HP1α and α-ISWI antibodies. Myc-Arp6, P18^Hamlet^, H2A and HP1α are present in the V5-CFDP1 immunoprecipitates, while they are absent from the immunoprecipitates of Ms IgG control. The HP1α present in V5 immunoprecipitates, although of weak intensity, has been detected in two independent experiments. HA-SRCAP and HA-H2A.Z has not been detected.

As shown in Fig. 5, Myc-Arp6, P18^Hamlet^ and H2A were detected in V5-CFDP1 immunoprecipitates, but were absent in the Ms IgG control. By contrast, HA-H2A.Z and HA-SRCAP were not detected in the IP. These results suggest that CFDP1 interacts with Arp6 and P18^Hamlet^ which are known members of the SRCAP complex.

Independent experimental evidence has shown that YETI interacts with HP1α in *Drosophila melanogaster*^24^,^33^. Given that, we also analyzed V5-CFDP1 immunoprecipitates using antibodies against HP1α. A reproducible HP1α band, although of weak intensity, was apparent in the IP sample and absent in the control (Fig. 5), suggesting that the interaction between BCNT proteins and HP1α is maintained during evolution.

## Discussion

### CFDP1 is required for chromatin organization in human cells

We have carried out the first *in vivo* functional characterization of the *Cfdp1* gene and its encoded protein in human cells. Two different isoforms of the protein can be detected in HeLa, U2OS and MRC5 cell lines (Fig. 1A), which most likely result from alternative splicing. Both bands are the result of *Cfdp1* expression, since their intensity strongly decreased following RNAi-mediated depletion of this gene (Fig. 1F,G).

Using IFM and chromatin fractionation we determined that CFDP1 has a nuclear localization and is found in the chromatin fraction in both interphase and metaphase (Figs 1 and 2). We also found that the chromatin-binding function of CFDP1 requires both its N- and C- terminal regions (Fig. 2). The N-terminal fragment used in our experiments lacks the last 82 amino acids as does the 35 kDa isoform. This region corresponds to the evolutionarily conserved BCNT-domain that was found to be crucial for the chromatin-binding activity of YETI in *D. melanogaster*^24^. We speculate that the 50 kDa isoform of CFDP1 is the one that participates in chromatin organization, while the 35 kDa isoform may have a different and still undetected function.

The results of co-IP assays (Fig. 5) indicated that in HeLa cells CFDP1 interacts with members of the SRCAP chromatin remodeling complex that exchanges H2A and H2A.Z histones (22). We also found an interaction with endogenous H2A, but not with HA-H2A.Z (Fig. 5). This finding suggests that CFDP1, by interacting with H2A, may play a role in an H2A-H2B eviction reaction, rather than in H2A.Z-H2B deposition.

In the light of these results, it is conceivable that CFDP1 bounds the chromatin through interaction with members of the SRCAP complex (Fig. 5) and that the N- and C- terminally truncated variants are unable to enter the complex. However, it cannot be completely ruled out that CFDP1 may also have an autonomous chromatin-binding activity independent of its interaction with the SRCAP complex.

RNAi-mediated depletion of CFDP1 led to mitotic chromosomes with aberrant morphology and condensation defects as well as chromosome segregation defects such as chromatin bridges (Fig. 3). These defects recall those exhibited by *Yeti* null alleles in *Drosophila melanogaster*^24^,^34^. Interestingly, the amount of SMC2 condensin, a major player in chromosome condensation that localizes to the axes of metaphase chromosomes, was strongly reduced in a high percentage of CFDP1-depleted cells (Fig. 3D,L,M,E). This could be a secondary consequence of drastic chromatin perturbations triggered by CFDP1 depletion. Alternatively, CFDP1 may play a direct role in the recruitment of condensin.

A recent study in fission yeast suggests that nucleosome eviction promotes condensin loading in mitosis^35^. This is interesting in view of the interaction we detected between CFDP1 and members of the SRCAP complex. In fact, both H2A and H2A.Z histones have also been proposed to be involved in condensin binding to chromatin^36^,^37^.

Moreover, the co-IP assays indicate that CFDP1 interacts with HP1α, in the same way that Drosophila YETI interacts with HP1a^24^,^33^. This finding suggests that BCNT proteins may be evolutionarily conserved mediators involved in the targeting of HP1 to chromatin remodeling regions.

We also found that *Drosophila* Flag-YETI expressed in HeLa cells enters the cell nucleus (Fig. 4A–C), binds to chromatin (Fig. 4D) and produces a strong decrease in the mitotic index (Fig. 4E), with an impairment of cell cycle progression. This could be due to the *in vivo* formation of inactive YETI-CFDP1 heterodimers (Fig. 4F), a result that corroborates our previous *in vitro* findings^29^.

### CFDP1 and craniofacial development

Craniofacial malformations are developmental disorders of crucial biomedical and clinical interest, since they represent the main cause of infant mortality and disability in humans. Together with cognitive defects and growth abnormalities, craniofacial malformations are common symptoms of epigenetic diseases^6^ and most known candidate genes are transcription factors and chromatin regulators^38^,^39^,^40^.

Among craniofacial diseases, autosomal recessive primary microcephaly (MCPH) is a rare disorder characterized by a reduction in brain size and head circumference at birth and mild to severe mental retardation. Thus far, mutations in 12 genes have been found in patients with MCPH; these genes affect cell cycle regulation and DNA repair^41^,^42^. Intriguingly, the *MCPH1* gene encoding microcephalin^38^ shows some aspects in common with *Cfdp1*. Microcephalin was found to regulate one of the two condensin complexes present in the cell, condensin II^43^. Similar to cells lacking CFDP1, *MCPH1*-depleted cells display aberrant chromosome condensation^44^. It is unclear whether this is the major pathological mechanism in patients with *MCPH1* mutations. Additional functions of MCPH1 during chromosome shaping and dynamics may be influenced^45^. Similarly, depletion of CFDP1 may affect other processes in addition to mitosis. Given its interaction with the SRCAP chromatin remodeler, the function of CFDP1 may affect both higher-order chromatin organization throughout the cell cycle and gene regulation. In any case, in view of the similarities with *MCPH1*, it would be interesting to include *Cfdp1* gene in the sequencing panels for microcephaly and related human disorders.

In conclusion, our findings provide new insight into the functions and mechanisms of the CFDP1 protein and contribute to our understanding of the link between epigenetic regulation and the onset of human craniofacial disorders.

## Methods

### Cytology and immunostaining

To analyze chromosome condensation, HeLa cells were treated at 37 °C for 2 h with 0,1 μg/mL colcemid, harvested by trypsinization and treated with hypotonic buffer (0,075 M KCl) for 10 min at room temperature. Cells were fixed with freshly made Fix solution (3:1 methyl alcohol:glacial acetic acid) at −20 °C. Then, chromosome preparations were made by air-drying method. For IF staining, HeLa cells were seeded on glass coverslips and 24 h later they were fixed for 15′ at room temperature (RT) in 2% formaldehyde in PBS. Cells were treated with hypotonic solution (75 mM KCl) for 30′ at RT before fixation. After permeabilization in 0.2% Triton X-100 solution and washing in PBS, the cells were incubated in 3% bovine serum albumin (BSA) for 1 h and, subsequently, with primary antibodies for 2 h at RT. After several washes in PBS, the cells were incubated with secondary antibodies for 1 h at RT. After washing in PBS, the coverslips were mounted onto slides with anti-FADE solution containing the anti-bleaching agent DABCO (Sigma-Aldrich). Preparations were analyzed using a computer-controlled Nikon Eclipse 50i epifluorescence microscope equipped with a CCD camera. CFDP1 fluorescence intensity was assessed using the ImageJ software (http://rsbweb.nih.gov/ij/). About 400 nuclei were scored in three independent experiments. The primary antibodies were: mouse monoclonal anti-CFDP1 (1:100; Sigma-Aldrich); rabbit polyclonal anti-CFDP1 (1:200; Thermo Scientific); rabbit polyclonal anti-hSMC2^46^; and rabbit anti-beta Tubulin (1:4000, Loading Control, Abcam). The secondary antibodies were: Donkey anti-rabbit IgG-CFL 555 (Santa Cruz Biotechnology; 1:200); and goat anti-mouse IgG-CFL 488 (Santa Cruz Biotechnology; 1:200).

### CFDP1 constructs

To make Flag-CFDP1 constructs, sequences corresponding to full length (Flag-CFDP1), N-terminal (Flag-CFDP1-Nt) and C-terminal/BCNT (Flag-CFDP1-Ct) of CFDP1 cDNA were cloned into the pENTR/D-TOPO (Invitrogen) and subcloned into pIRESpuro3-MycFlag/GATCassette Gateway destination vector. V5-CFDP1 and Flag-YETI plasmid are described in our previous papers^24^,^29^. The constructs pCS2-5xMyc-Arp6, pcDNA3-HA-H2A.Z and pcDNA3-HA-SRCAP were described in ref. 47. For a description of the CFDP1 constructs, see Fig. S1.

### Cell Cultures and RNAi treatments

HeLa cells, purchased from ATTC company, were cultured in 6-well plates in Dulbecco’s modified Eagle’s medium (DMEM) supplemented with 10% FBS and a Penicillin/Streptomycin solution (Gibco, 15140122). Transfection was performed with 1 μg of CFDP1 esiRNA using Dharmafect (Thermo Scientific) according to the manufacturer’s protocol. Endoribonuclease-prepared siRNA (esiRNA) that targets CFDP1 was purchased from Sigma-Aldrich. Two days after transfection cells were harvested for cytological and immunoblotting analysis.

### Cell cycle Synchronization

For cell cycle-dependent chromatin binding experiments, HeLa cells were synchronized in interphase (G1/S boundary) or metaphase using thymidine or thymidine/nocodazole blocks, respectively. Briefly, for G1/S boundary synchronization, cells were treated with 2 mM thymidine (Sigma, T9250) for 19 h, washed with PBS, harvested by centrifugation and frozen in liquid nitrogen. For metaphase synchronization, after thymidine treatment, HeLa cells were released from G1/S block in fresh media for 5 h, incubated with 40 nM nocodazole (Sigma, M1403) for 16 h and then harvested by mitotic shake-off. Mitotic cells were washed three times with PBS and released in fresh medium for 30′ before harvesting and freezing in liquid nitrogen. Interphase and metaphase cell samples were prepared by resuspending cells in Buffer A for subsequent chromatin fractionation assay.

### Chromatin Fractionation Assay

HeLa cells (2 × 10^7^) were resuspended in 1 mL of Buffer A (10 mM HEPES at pH 7.9, 10 mM KCl, 1.5 mM MgCl_2_, 0.34 M sucrose, 10% glycerol, 1 mM dithiothreitol [DTT], 1 mM NaVO_4_, 5 mM β-glycero-phosphate, 0.1 mM phenyl methane sulphonyl fluoride [PMSF], 0.5 mM NaF, protease inhibitor cocktail [Roche]). Triton X-100 was added at 0.1% and cells were incubated for 5′ on ice. After centrifugation (4000 rpm for 5′ at 4 °C), the nuclei enriched pellet was resuspended in 1 mL of Buffer B (3 mM EDTA, 0.2 mM EGTA, 1 mM DTT, 0.1 mM PMSF, protease inhibitors) and incubated for 5′ on ice. All the fractioned samples were load in a polyacrylamide gel and transferred onto PVDF membrane for Western blot analysis.

### Immunoprecipitations

HeLa cells were transfected with 2 μg of V5-Cfdp1 and Myc-Arp6 or HA-H2A.Z expression vectors by using Lipofectamine 3000 reagent according to the manufacture’s protocol, and 48 h later, they were lysated in IP buffer (50 mM Tris–HCl pH 7.5, 150 mM NaCl, 1% NP-40, 5 mM EGTA, 5 mM EDTA, 20 mM NaF) supplemented with protease and phosphatase inhibitors cocktail (from Roche) and 1 mM PMSF. Cleared lysates were immunoprecipitated O/N at 4 °C with 2 μg of mouse anti-V5 antibodies (Invitrogen) followed by 1 h of incubation with 30 μl of agarose-conjugated protein A/G (Santa Cruz Biotechnology). The beads were then washed three times in IP buffer and analyzed by immunoblotting.

### Western blotting

HeLa cells were lysated in sample buffer at 10000 cells/μl. All the samples were load in a polyacrilamyde gel, transferred onto PVDF membrane and probed with different antibodies: mouse monoclonal anti-CFDP1 (1:1000; Sigma-Aldrich); rabbit polyclonal anti-CFDP1 (1:1000, Thermo Scientific); mouse monoclonal anti-FLAG (1:1000; Sigma-Aldrich); mouse monoclonal anti-HA (1:1000, Cell Signaling); mouse monoclonal anti-c-Myc (1:1000, Clontech); mouse monoclonal anti-V5 (1:1000, Thermo Scientific); rabbit polyclonal anti-HP1α (1:1000, Cell Signaling); rabbit polyclonal anti-histone H2A (1:1000, Millipore); rabbit polyclonal anti-p18^Hamlet^ (1:200)^48^; rabbit polyclonal anti-MEK2 (1:1000, Santa Cruz Biotechnology); rabbit polyclonal anti-histone H3 (1:15000, Abcam); rabbit polyclonal anti-H3p (1:5000)^49^; rabbit polyclonal anti-ISWI (1:5000)^50^. The bands were immunodetected using the Enhanced chemiluminescence (ECL) kit from Thermo Scientific.

### Statistical analysis

Data analyses were performed using the GraphPad Prism softwares (GraphPad Software, Inc., La Jolla, CA, USA). All results are expressed as mean ± SD values from three independent replicate experiments. *P* value of less than 0.05 (*P < 0.05, compared with the control group) are considered to be statistically significant by using two-tailed Fisher’s exact test.

### Ethical approval and informed consent

All the methods were carried out in accordance with the approved guidelines.

## Additional Information

**How to cite this article:** Messina, G. *et al*. The human *Cranio Facial Development Protein 1 (Cfdp1)* gene encodes a protein required for the maintenance of higher-order chromatin organization. *Sci. Rep.* **7**, 45022; doi: 10.1038/srep45022 (2017).

**Publisher's note:** Springer Nature remains neutral with regard to jurisdictional claims in published maps and institutional affiliations.

## Supplementary Material

Supplementary Figure s1

## References

[b1] Clapier C. R. & Cairns B. R. The biology of chromatin remodeling complexes. Annual review of biochemistry 78, 273–304, doi: 10.1146/annurev.biochem.77.062706.153223 (2009). 19355820

[b2] Gilbert N. . Formation of facultative heterochromatin in the absence of HP1. The EMBO journal 22, 5540–5550, doi: 10.1093/emboj/cdg520 (2003). 14532126 PMC213774

[b3] Fog C. K., Galli G. G. & Lund A. H. PRDM proteins: important players in differentiation and disease. BioEssays: news and reviews in molecular, cellular and developmental biology 34, 50–60, doi: 10.1002/bies.201100107 (2012). 22028065

[b4] Bouazoune K. & Kingston R. E. Chromatin remodeling by the CHD7 protein is impaired by mutations that cause human developmental disorders. Proceedings of the National Academy of Sciences of the United States of America 109, 19238–19243, doi: 10.1073/pnas.1213825109 (2012). 23134727 PMC3511097

[b5] Masliah-Planchon J., Bieche I., Guinebretiere J. M., Bourdeaut F. & Delattre O. SWI/SNF chromatin remodeling and human malignancies. Annual review of pathology 10, 145–171, doi: 10.1146/annurev-pathol-012414-040445 (2015). 25387058

[b6] Brookes E. & Shi Y. Diverse epigenetic mechanisms of human disease. Annual review of genetics 48, 237–268, doi: 10.1146/annurev-genet-120213-092518 (2014). 25195505

[b7] Kumar R., Li D. Q., Muller S. & Knapp S. Epigenomic regulation of oncogenesis by chromatin remodeling. Oncogene, doi: 10.1038/onc.2015.513 (2016). 26804164

[b8] Watrin E., Kaiser F. J. & Wendt K. S. Gene regulation and chromatin organization: relevance of cohesin mutations to human disease. Current opinion in genetics & development 37, 59–66, doi: 10.1016/j.gde.2015.12.004 (2016). 26821365

[b9] Messina G., Atterrato M. T. & Dimitri P. When chromatin organisation floats astray: the Srcap gene and Floating-Harbor syndrome. Journal of medical genetics, doi: 10.1136/jmedgenet-2016-103842 (2016). 27208210

[b10] Vissers L. E. . Mutations in a new member of the chromodomain gene family cause CHARGE syndrome. Nature genetics 36, 955–957, doi: 10.1038/ng1407 (2004). 15300250

[b11] Basson M. A. & van Ravenswaaij-Arts C. Functional Insights into Chromatin Remodelling from Studies on CHARGE Syndrome. Trends in genetics: TIG 31, 600–611, doi: 10.1016/j.tig.2015.05.009 (2015). 26411921 PMC4604214

[b12] Iwashita S. a. O., N. In Gene Duplication Ch. 21, 383–400 (2011).

[b13] Messina G. . The Bucentaur (BCNT) protein family: a long-neglected class of essential proteins required for chromatin/chromosome organization and function. Chromosoma 124, 153–162, doi: 10.1007/s00412-014-0503-8 (2015). 25547403

[b14] Iwashita S. . Mammalian Bcnt/Cfdp1, a potential epigenetic factor characterized by an acidic stretch in the disordered N-terminal and Ser250 phosphorylation in the conserved C-terminal regions. Bioscience reports 35, doi: 10.1042/BSR20150111 (2015). PMC4613681 26182435

[b15] Diekwisch T. G., Marches F., Williams A. & Luan X. Cloning, gene expression, and characterization of CP27, a novel gene in mouse embryogenesis. Gene 235, 19–30 (1999). 10415329 10.1016/s0378-1119(99)00220-6

[b16] Diekwisch T. G., Luan X. & McIntosh J. E. CP27 localization in the dental lamina basement membrane and in the stellate reticulum of developing teeth. The journal of histochemistry and cytochemistry: official journal of the Histochemistry Society 50, 583–586 (2002). 11897812 10.1177/002215540205000416

[b17] Thisse B. . Spatial and temporal expression of the zebrafish genome by large-scale *in situ* hybridization screening. Methods in cell biology 77, 505–519 (2004). 15602929 10.1016/s0091-679x(04)77027-2

[b18] Wu M. . Persistent expression of Pax3 in the neural crest causes cleft palate and defective osteogenesis in mice. The Journal of clinical investigation 118, 2076–2087, doi: 10.1172/JCI33715 (2008). 18483623 PMC2381747

[b19] Bustos-Valenzuela J. C., Fujita A., Halcsik E., Granjeiro J. M. & Sogayar M. C. Unveiling novel genes upregulated by both rhBMP2 and rhBMP7 during early osteoblastic transdifferentiation of C2C12 cells. BMC research notes 4, 370, doi: 10.1186/1756-0500-4-370 (2011). 21943021 PMC3196718

[b20] Makeyev A. V. & Bayarsaihan D. Molecular basis of Williams-Beuren syndrome: TFII-I regulated targets involved in craniofacial development. The Cleft palate-craniofacial journal : official publication of the American Cleft Palate-Craniofacial Association 48, 109–116, doi: 10.1597/09-093 (2011). 20500075

[b21] Havugimana P. C. . A census of human soluble protein complexes. Cell 150, 1068–1081, doi: 10.1016/j.cell.2012.08.011 (2012). 22939629 PMC3477804

[b22] Monroy M. A. . Regulation of cAMP-responsive element-binding protein-mediated transcription by the SNF2/SWI-related protein, SRCAP. The Journal of biological chemistry 276, 40721–40726, doi: 10.1074/jbc.M103615200 (2001). 11522779

[b23] Mizuguchi G. . ATP-driven exchange of histone H2AZ variant catalyzed by SWR1 chromatin remodeling complex. Science 303, 343–348, doi: 10.1126/science.1090701 (2004). 14645854

[b24] Messina G. . Yeti, an essential Drosophila melanogaster gene, encodes a protein required for chromatin organization. Journal of cell science 127, 2577–2588, doi: 10.1242/jcs.150243 (2014). 24652835

[b25] Kusch T. . Acetylation by Tip60 is required for selective histone variant exchange at DNA lesions. Science 306, 2084–2087, doi: 10.1126/science.1103455 (2004). 15528408

[b26] Wu W. H. . N terminus of Swr1 binds to histone H2AZ and provides a platform for subunit assembly in the chromatin remodeling complex. The Journal of biological chemistry 284, 6200–6207, doi: 10.1074/jbc.M808830200 (2009). 19088068 PMC2649089

[b27] Morillo-Huesca M., Clemente-Ruiz M., Andujar E. & Prado F. The SWR1 histone replacement complex causes genetic instability and genome-wide transcription misregulation in the absence of H2A.Z. PloS one 5, e12143, doi: 10.1371/journal.pone.0012143 (2010). 20711347 PMC2920830

[b28] Baldi S. & Becker P. B. The variant histone H2A.V of Drosophila-three roles, two guises. Chromosoma 122, 245–258, doi: 10.1007/s00412-013-0409-x (2013). 23553272

[b29] Messina G., Atterrato M. T., Fanti L., Giordano E. & Dimitri P. Expression of human Cfdp1 gene in Drosophila reveals new insights into the function of the evolutionarily conserved BCNT protein family. Scientific reports 6, 25511, doi: 10.1038/srep25511 (2016). 27151176 PMC4858687

[b30] Rual J. F. . Towards a proteome-scale map of the human protein-protein interaction network. Nature 437, 1173–1178, doi: 10.1038/nature04209 (2005). 16189514

[b31] Hock R., Scheer U. & Bustin M. Chromosomal proteins HMG-14 and HMG-17 are released from mitotic chromosomes and imported into the nucleus by active transport. The Journal of cell biology 143, 1427–1436 (1998). 9852141 10.1083/jcb.143.6.1427 PMC2132996

[b32] Hirano T. Condensins: universal organizers of chromosomes with diverse functions. Genes & development 26, 1659–1678, doi: 10.1101/gad.194746.112 (2012). 22855829 PMC3418584

[b33] Ryu H. W. . Analysis of the heterochromatin protein 1 (HP1) interactome in Drosophila. Journal of proteomics 102, 137–147, doi: 10.1016/j.jprot.2014.03.016 (2014). 24681131

[b34] Cenci G., Belloni G. & Dimitri P. 1(2)41Aa, a heterochromatic gene of Drosophila melanogaster, is required for mitotic and meiotic chromosome condensation. Genetical research 81, 15–24 (2003). 12693679 10.1017/s0016672302006018

[b35] Toselli-Mollereau E. . Nucleosome eviction in mitosis assists condensin loading and chromosome condensation. The EMBO journal 35, 1565–1581, doi: 10.15252/embj.201592849 (2016). 27266525 PMC4946138

[b36] Tada K., Susumu H., Sakuno T. & Watanabe Y. Condensin association with histone H2A shapes mitotic chromosomes. Nature 474, 477–483, doi: 10.1038/nature10179 (2011). 21633354

[b37] Kim H. S. . An acetylated form of histone H2A.Z regulates chromosome architecture in Schizosaccharomyces pombe. Nature structural & molecular biology 16, 1286–1293, doi: 10.1038/nsmb.1688 (2009). PMC2788674 19915592

[b38] Cohen M. M. Jr. Malformations of the craniofacial region: evolutionary, embryonic, genetic, and clinical perspectives. American journal of medical genetics 115, 245–268, doi: 10.1002/ajmg.10982 (2002). 12503119

[b39] Pallares L. F. . Mapping of Craniofacial Traits in Outbred Mice Identifies Major Developmental Genes Involved in Shape Determination. PLoS genetics 11, e1005607, doi: 10.1371/journal.pgen.1005607 (2015). 26523602 PMC4629907

[b40] Woods C. G., Bond J. & Enard W. Autosomal recessive primary microcephaly (MCPH): a review of clinical, molecular, and evolutionary findings. American journal of human genetics 76, 717–728, doi: 10.1086/429930 (2005). 15806441 PMC1199363

[b41] Bond J. . ASPM is a major determinant of cerebral cortical size. Nature genetics 32, 316–320, doi: 10.1038/ng995 (2002). 12355089

[b42] Faheem M. . Molecular genetics of human primary microcephaly: an overview. BMC medical genomics 8 Suppl 1, S4, doi: 10.1186/1755-8794-8-S1-S4 (2015). PMC4315316 25951892

[b43] Yamashita D. . MCPH1 regulates chromosome condensation and shaping as a composite modulator of condensin II. The Journal of cell biology 194, 841–854, doi: 10.1083/jcb.201106141 (2011). 21911480 PMC3207293

[b44] Trimborn M., Schindler D., Neitzel H. & Hirano T. Misregulated chromosome condensation in MCPH1 primary microcephaly is mediated by condensin II. Cell cycle 5, 322–326, doi: 10.4161/cc.5.3.2412 (2006). 16434882

[b45] Arroyo M. . Chromosome structure deficiencies in MCPH1 syndrome. Chromosoma 124, 491–501, doi: 10.1007/s00412-015-0512-2 (2015). 25845520

[b46] Kimura K., Cuvier O. & Hirano T. Chromosome condensation by a human condensin complex in Xenopus egg extracts. The Journal of biological chemistry 276, 5417–5420, doi: 10.1074/jbc.C000873200 (2001). 11136719

[b47] Cuadrado A. . Essential role of p18Hamlet/SRCAP-mediated histone H2A.Z chromatin incorporation in muscle differentiation. The EMBO journal 29, 2014–2025, doi: 10.1038/emboj.2010.85 (2010). 20473270 PMC2892367

[b48] Cuadrado A. . A new p38 MAP kinase-regulated transcriptional coactivator that stimulates p53-dependent apoptosis. The EMBO journal 26, 2115–2126, doi: 10.1038/sj.emboj.7601657 (2007). 17380123 PMC1852783

[b49] Kimura K. & Hirano T. Dual roles of the 11S regulatory subcomplex in condensin functions. Proceedings of the National Academy of Sciences of the United States of America 97, 11972–11977, doi: 10.1073/pnas.220326097 (2000). 11027308 PMC17279

[b50] MacCallum D. E., Losada A., Kobayashi R. & Hirano T. ISWI remodeling complexes in Xenopus egg extracts: identification as major chromosomal components that are regulated by INCENP-aurora B. Molecular biology of the cell 13, 25–39, doi: 10.1091/mbc.01-09-0441 (2002). 11809820 PMC65070

