# Supplementary material for: The human Cranio Facial Development Protein 1 (Cfdp1) gene encodes a protein required for the maintenance of higher-order chromatin organization
Source: Sci Rep. 2017 Apr 3;7:45022. doi: 10.1038/srep45022 (PMC5377257; doi:10.1038/srep45022)
Supplement: Supplementary Figure s1 [file srep45022-s1.pdf]

# The human *Cranio Facial Development Protein 1* (*Cfdp1*) gene encodes a protein required for the maintenance of higher-order chromatin organization

**Giovanni Messina[1,2], Maria Teresa Atterrato[1,2], Yuri Prozzillo [1,2], Lucia Piacentini[2], Ana Losada[3] and Patrizio Dimitri[1,2]**

[1]Istituto Pasteur Italia, Fondazione Cenci-Bolognetti and [2]Dipartimento di Biologia e Biotecnologie "Charles Darwin" Sapienza Università di Roma, Roma, Italy; [3]CNIO, Madrid, Spain

## Supplementary materials

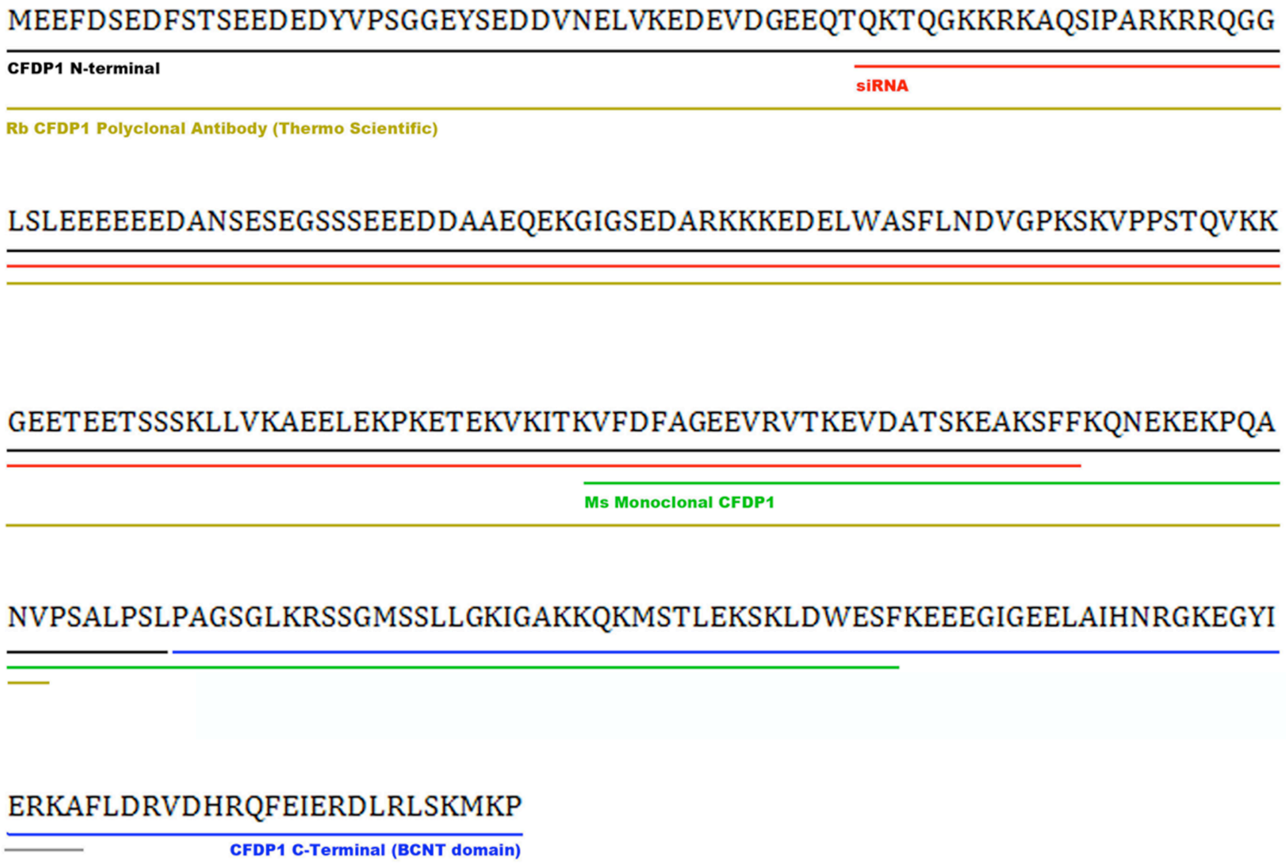


## Figure S1

Scheme showing the full-length CFDP1 amino acid sequence (299 amino acids) where the FLAG-tagged proteins, antibody epitopes and siRNA target sequences are underscored. The Flag-CFDP1-Nt/Isoform 2 (1–217) is shown in black; Flag-CFDP1-Ct/BCNT domain (218–299) in blue; the epitope recognized by mouse monoclonal anti-CFDP1(Sigma-Aldrich) and rabbit polyclonal anti-CFDP1 (Thermo Scientific) are shown in green and ochre yellow, respectively. A siRNA (Sigma-Aldrich) used for *Cfdp1* depletion targets the *Cfdp1* mRNA sequence encoding the amino acid portion shown in red.
